# Health inequality in the Russian Federation: An examination of the changes in concentration and achievement indices from 1994 to 2013

**DOI:** 10.1186/s12939-016-0325-9

**Published:** 2016-02-29

**Authors:** Pavitra Paul, Hannu Valtonen

**Affiliations:** Department of Health and Social Management, Faculty of Social Sciences and Business Studies, University of Eastern Finland, Kuopio, 70211 Finland

**Keywords:** Achievement, Aversion, Concentration, Distributionally-sensitive, Health inequalities, Positive-externality, Self-perceived, Subjective socioeconomic status, Russia

## Abstract

**Background:**

To assess and quantify the magnitude of health inequalities ascribed to socioeconomic strata from 1994 to 2013 in the Russian Federation.

**Methods:**

A balanced sample of 1,496 adult individuals extracted from the 1994 wave of the Russian Longitudinal Monitoring Survey (RLMS) is followed for stated self-perceived health status until 2013. The socioeconomic strata (SES) index is constructed with a set of variables (adult equivalent household income, ownership of assets and living conditions) by applying principal component analysis (PCA). We use a regression-based concentration index to measure differences in self-perceived health status. Finally, we examine the degree of aversion to inequalities in self-perceived health status between the worse-off and the better-off with the achievement index.

**Results:**

By 2013, the mean standardized self-perceived health status has improved by 4.6 % compared to 1994. The absolute size of Concentration Index (CI) for non - standardized self-perceived health status is reduced by 44.27 % from 1994 to 2013. No systematic trend emerges in the evolution of CI for self-perceived health status of the Russians over the 19 year period. However, avoidable inequalities in self-perceived health status of the Russian population is reduced by almost 60 % over the two decades (1994–2013).

**Conclusion:**

SES, as defined with objective indicators, shows little consistency in association with self-perceived health status in the Russian Federation. This study highlights the need for future research that considers the context of stated self-perceived health status in the realm of subjective socioeconomic status (SSS).

## Background

The collapse of the Soviet Union resulted in large fluctuations in household resources and increased uncertainty for the Russian people. Much academic research has explored the socioeconomic and political ramifications of this transition to a mainstream market economy. The importance of health in times of rapid change in the economy has been highlighted in economic development literature [1, 2]. Preston [3] concludes that factors other than individual income play a powerful role in the determinants of health conditions that are often associated with poverty. This reinforces the idea that individual incomes in isolation from the community in which people are located do not immediately reflect health status. Neighbourhood affluence is a more powerful predictor of health status than poverty, above and beyond individual demographic background, socioeconomic status, and health behaviours [4]. An empirical study using Spanish Living Conditions Survey datasets (2005–2008) revealed that the relationship between health and income operates through comparison information with respect to societal peers; conversely, material deprivation in terms of financial difficulties, basic necessities, and housing conditions exerts a direct effect on one’s self-perceived health status [5].

Vella [6] has reported the prevalence of a considerably high share of dissatisfaction with self-perceived health status among the respondents of the Russian Longitudinal Monitoring Survey, 1993, and Cockerham [7] has found no relationship between income and self-perceived health status among the respondents of the Russian Longitudinal Monitoring Survey, 1995. Another cross-sectional study by Cockerham et al. [8] reported a similar relationship between income and self-perceived health status among the respondents of the Russian Longitudinal Monitoring Survey, 1998. Further, the Russian Longitudinal Monitoring Survey, 1998 reflected a strong negative correlation between the health status of the individual and the desire to return to the former Soviet regime – a reflection of dissatisfaction with the economic and social conditions of the time [8]. A panel data frame study using Russian Longitudinal Monitoring Survey (1994–2000) datasets showed little effect of income on self-perceived health status when the possibilities of reverse causation and incidental association between income and health were accounted for (Stepanyan A. Is Economic Transition a Health Hazard? Self-perceived Health and Income in Russia: 1994–2000. Unpublished working paper 2002). However, a longitudinal study (Jensen RT. Job Security, Stress and Health: Evidence From Russian Privatization. Unpublished working paper 2005) using Russian Longitudinal Monitoring Survey datasets (1992–1994) suggested a correlation between negative health consequences with concern about the likelihood of job loss.

Kraus et al. [9] have shown that chronically experienced negative affects (higher temperamental sensitivity to negative stimuli resulting in feelings of fear, anxiety, depression, guilt, and self-dissatisfaction) explains rather than confounds the link between socioeconomic strata and self-perceived health. The systematic review study of Ostrove and Adler [10] suggests that the pathway from socioeconomic strata to health is through exposure to different environments and adaptations to these environments. Environmental demands and supports shape individual responses to self-perceived health. Unemployment and inflation have often been considered two of the most robust determinants of individual happiness, with unemployment typically having a stronger negative impact than inflation. Eggers et al. [11] found that in the Russian Federation, higher unemployment rates lead to more happiness, as people tend to revise their expectations upwards when their neighbours are suffering. Rodríguez-Pose and Maslauskaite [12] argued that citizens are quite tolerant of interpersonal inequality, and as the economy is still dynamic, open and volatile, the influence of macroeconomic factors on happiness varies substantially over time; therefore cross-sectional analysis cannot provide any robust conclusions.

An empirical study (using eight waves of the European Community Household Panel) reflects a larger effect of activity status on self-perceived health than the effect of income. Inactivity is associated with the largest absolute effect, in part, among individuals claiming disability allowances due to ill health [13]. A cross-sectional study [14] on the evolution of health inequalities in Switzerland found no systematic trend in the changes with the measure of health inequalities between 1982 and 2002.

The ecosocial theory of health distribution, introduced by Krieger in 1994 [15] postulates that (1) population health and health inequities must be analysed in a societal, historical, and ecological context, and (2) neither the forms of social inequality nor their associations with health status are “fixed”, but are instead historically contingent. The relationship between socioeconomic strata (SES) and health changes with age, since the mediators of SES act differently in different stages of the life course. Health in later life is the result of multiple social and biological processes whose effects interact and accumulate over time [16–18].

The microeconomic perspective assesses health costs (or the economic benefits of good health) at the individual or household level. Macroeconomic consequences are viewed from the level of the national economy, generally considering the level of population health that retards a country’s economic growth. The realization of the economic objectives with a concurrent reduction of poverty (more relevant for transitional economies) necessitates evidence-informed policy development for investment in health. The micro perspective is also specifically important for individuals and households, as freedom from ill health adds different dimensions of economic well-being to individuals and households. Further, the concern for poverty and inequality recognizes that in health status societal averages typically disguise as much as they reveal, therefore, the interest is not in health status that prevail in society as a whole but in health status of different socioeconomic groups [19].

This paper investigates how the difference in the self-perceived health status of Russians has evolved in the context of the transition to a mainstream market economy, and following the approach of Wagstaff, Makinen et al. and Castro-Leal et al. [20–22], this study examines the trend in the distribution of such difference across the socioeconomic strata of the population from 1994 to 2013. Our data is unique in the sense that we follow the same individuals over an 18-year period.

## Methods

The data for this study are from the 1994–2013 waves of the Russian Longitudinal Monitoring Survey (RLMS). RLMS (http://www.cpc.unc.edu/rlms) is an on-going longitudinal household survey of the Russian Federation. The survey was designed to be representative of the Russian population in the early 1990s. The survey contains an array of information on the economic, social, demographic, and health characteristics of respondents, their households, and the environments in which they live. An initial representative sample of dwellings was drawn in 1992 and surveyed every six months until 1994 (phase 1). A second more broadly drawn representative sample (phase 2) replaced the initial sample in 1994 and is being followed annually since then. For cost reasons, RLMS does not attempt to follow individuals or households who move from their original sample dwelling units (attempts are made to follow individuals and households that move locally); instead, any new household member or household living at that dwelling is included in the sample in each wave. Thus, the RLMS sample remains representative of the underlying population if new residents can be considered exchangeable with those that moved.^1^

The RLMS data sets contain post-stratification weights, that is, weights that adjust not only for design factors but also for deviations from census characteristics. Variation in individual weights reflects the geographic effects for households as well as differentials due to post-stratification of the sample by major geographic region, age, and gender. The multivariate distribution of the sample by gender, age, and urban/rural location compared quite well with the corresponding multivariate distribution of the 1989 census (http://www.cpc.unc.edu/rlms).

From this unbalanced sample, we chose RLMS participants who had been included in all 18 waves. Thus, after balancing the panel, we followed 1,496 individuals (Table 1), henceforth to be read as study sample. We explored the study sample selection (i.e., difference in characteristics of respondents between the extracted individual and RLMS – 1994 datasets) with the binary (study sample = 1, RLMS – 1994 datasets = 0) using logit model. Hence, with the long (19 year) follow-up of the individuals, we have controlled the variations attributed to the sampling population per se. The use of more than one observation facilitates causal inference in situations where inferring causality becomes very hard if only a single cross section are used and the panel nature of the data allows for the unobserved effect to be correlated with the explanatory variables – thus, ensures a better control for the unobserved effects of the variables [23].

**Table 1:** Descriptive statistics

	2013 *(N = 1496)*	1994 (*N* = 1496)	RLMS data - 1994 (*N = 8893*)	Test of independence (between the datasets - 1994 and extracted sample)
Variables				
Age group (in years)				
<30		24.53	28.8	
31- 44		37.83	25.97	
45 - 60		29.75	21.7	
61 - 74		7.69	17.32	
75+		0.2	6.21	
				*χ* ^2^, [*p*-value = 0.000]
Age group (in years) by gender		Female (%)	Female (%)	
<30		60.76	52.18	
31- 44		61.84	52.75	
45 - 60		71.69	56.15	
61 - 74		70.43	61.92	
75+		100.0	78.35	
Total		65.24	56.14	
				*χ* ^2^, [*p*-value = 0.000]
Settlement of residence (%)				
Urban	51.27	52.11	69.32	*χ* ^2^, [*p*-value = 0.000]
Self-perceived (self-assessed) health (%)				
Very Good	0.61	1.54	1.93	
Good	17.26	23.04	23.4	
Average	57.13	62.89	54.44	
Bad	21.94	10.98	16.52	
Very bad	3.06	1.54	3.7	
				*χ* ^2^, [*p*-value = 0.000]
Diagnosed Chronic disease^a^ (%)	16.38	7.29	8.46	*χ* ^2^, [*p*-value = 0.000]
Satisfaction with life^b^ (%)	72.47	31.18	35.40	*χ* ^2^, [*p*-value = 0.000]
Adult equivalent household income (Rb. per month inflation adjusted at the level of June 1992)				
Mean	7610	4110	5410	
Median	6880	3220	3760	
				*t*-test [*p*-value = 0.000]
Ownership of assets (%)				
Tractor	4.88	2.08	1.26	*χ* ^2^, [*p*-value = 0.002]
Motorvehicle	25.30	26.54	25.46	*χ* ^2^, [*p*-value = 0.296]
Country house (dacha)	18.65	12.03	13.87	*χ* ^2^, [*p*-value = 0.024]
Own house	93.65	83.87	87.88	*χ* ^2^, [*p*-value = 0.000]
Addl. Appartment	5.08	2.61	4.36	*χ* ^2^, [*p*-value = 0.000]
Living condition (% of respondents with access to public services utilities)				
Central heating	52.44	55.78	71.28	*χ* ^2^, [*p*-value = 0.000]
Hot water supply	47.86	42.63	57.45	*χ* ^2^, [*p*-value = 0.000]
Cooking gas	71.05	64.03	75.18	*χ* ^2^, [*p*-value = 0.000]
Central garbage disposal	55.72	52.16	67.11	*χ* ^2^, [*p*-value = 0.000]
Other indicators of economic status (%)				
Grow crops (incl. growing vegetables in house lawns and rented lands)	92.86	98.5	96.29	*χ* ^2^, [*p*-value = 0.000]
Having savings^c^	18.13	8.63	10.79	*χ* ^2^, [*p*-value = 0.003]
SES quintile^d^ (%)				
Poorest	20.10	20.06	20.06	
2nd poorest	19.94	20.05	19.97	
Middle	20.01	20.06	19.99	
2nd richest	20.01	20.05	20.06	
Richest	19.94	19.78	19.92	

^a^Diagnosed with and or suffered from Diabetes, heart attack, stroke and anaemia

^b^Response to the question: ’to what extent are you satisfied with your life in general at the present time? ’[1 = fully satisfied, rather satisfied and both yes and no; 0 = less than satisfied and not at all satisfied]

^c^Response to the question: ‘did your family in the last 30 days save any money?’

^d^Distribution of respondents in quintiles based the constructed socioeconomic strata

Our dependent variable for the analysis is self-perceived (self-perceived) health (SAH). Individuals were asked, “How would you evaluate your health?” and the response was recorded on a five-point Likert scale with the answers “very good”, “good”, “average – not good but not bad”, “bad”, and “very bad”. SAH variables have been widely used in literatures [24–27] that analyse the socioeconomic health gradient.

We constructed an SES index by applying principal component analysis (PCA) using a set of variables (adult equivalent household income, ownership of assets, living conditions, and other indicators of material affluence, i.e., having savings and growing crops) with a Kaiser-Meyer–Olkin score of 0.80 and above. With the RLMS – 1994, PCA resulted in two principal components (PC), first PC accounting for 29 % of the total variance captured in seven items (adult equivalent household income, country house, own house, additional apartment, central heating, hot water supply and use of cooking gas), while the second PC accounted for 15 %, captured in four items (adult equivalent household income, country house, own house and additional apartment). With the data sub-set (study sample), the first PC accounted for 33 % of the total variance captured in seven items (adult equivalent household income, country house, own house, additional apartment, central heating, hot water supply and use of cooking gas), while the second PC accounted for 15 %, captured in four items (adult equivalent household income, country house, additional apartment, central heating). The income variable represented the sum of incomes from all sources for the household deflated to the value of June 1992. We calibrated the household income as per adult equivalent using the modified OECD scale^2^ for our analyses. We measured inequality in income by the Gini index.^3^

We standardized [28, 29] the self-perceived health status by age, gender, and diagnosed chronic diseases, applying the indirect method of standardization.^4^ We estimated the correlation of the confounding variables (age, gender, and diagnosed chronic diseases) with self-perceived health status conditional on non-confounding variables (region, settlement of residence, and SES). This regression-based approach (Eqn. 1) “corrects” the actual distribution of the self-perceived health status by comparing it with the distribution that would be observed if all individuals in the group had their own age and gender characteristics but the same mean age and gender effect as the entire population.

1 $$ {y}_i=\alpha +{\displaystyle {\sum}_j{\beta}_j{\mathrm{x}}_{ji}}+{\displaystyle {\sum}_k{\gamma}_k{z}_{ki}+{\epsilon}_{i,}} $$

where, *y*_*i*_ is self-perceived health status; *i* denotes the individual; and α, β, and γ are parameter vectors. The x_*j*_ are confounding variables (age, gender, and diagnosed chronic diseases), which we want to standardize, and the *z*_*k*_ are non-confounding variables (region, settlement of residence, and SES), which we do *not* want to standardize but to control for in order to estimate partial correlations with the confounding variables. The Newey–West^5^ estimator estimates ($\widehat{\alpha},{\widehat{\beta}}_j,\ {\widehat{\gamma}}_k$) the individual values of the confounding variables (x_*ji*_), and sample means of the non-confounding variables (${\overline{z}}_k$) are then used to obtain the predicted, or “x-expected,” values of the self-perceived health status *ŷ*_*i*_^*x*^.

2 $$ {\widehat{y}}_i^x=\widehat{\alpha}+{\displaystyle {\sum}_j{\widehat{\beta}}_j{\mathrm{x}}_{ji}}+{\displaystyle {\sum}_k{\widehat{\gamma}}_k{\overline{z}}_k.} $$

Estimates of indirectly standardized self-perceived health:

3 $$ {\widehat{Y}}_i^{IS}={Y}_i\ \mathit{\hbox{-}}\ {\widehat{Y}}_i^X + \overline{Y} $$

where,

- *Ŷ*_*i*_^*IS*^ = *indirectly standardized, self-perceived health status*
- *Y*_*i*_ = *actual health*
- *Ŷ*_*i*_^*X*^ = *x-expected health*
- $\overline{Y}$ = *overall sample mean*

In the next step, following the principles of previous analyses [30, 31], we dichotomized the five-scaled measure into a binary variable [32], “self-perceived health” (1 = good, i.e., responded as “very good”, “good”, and 0 = not good, i.e., responded as “average”, “bad” and “very bad”).

The conventional regression-based statistical methods report the magnitude and the direction of association between socioeconomic position and the health status of the individual but ignores the possibility of variance in the effect of explanatory variables across distribution. Further, such traditional methods cannot reflect the extent of health disparity across socioeconomic strata of the population and thus do not allow for comparison over time [30]. Therefore, we used the health concentration index (Appendix: A) as our measure of SES-related inequality. The concentration index (CI) becomes positive if health (i.e., self-perceived health status) is concentrated amongst the better-off, negative if health (i.e., self-perceived health status) is concentrated amongst the worse-off, and zero if no inequality is observed. Thus, CI can also be interpreted as the slope of a line passing through the heads of an army of people, ranked by their SES, with the height for each individual proportionate to the value of his/her self-perceived health status, expressed as a fraction of the mean for the group.

Finally, with the achievement index^6^ (Appendix: B), we measured the average level of health and the inequality in health between the worse-off and the better-off. Achievement is the weighted average of the health (self-perceived) level of the individuals (members) in the study (community) where higher weights are attached to worse-off than to better-off (The mean is clearly not appropriate, since it weights everyone’s health, here, self-perceived health status equally, irrespective of how poor they are). Thus, achievement index is distributionally sensitive measure of population health. CI captures the extent to which ill health is concentrated amongst the worse-off. Achievement index indicates the degree of aversion to inequalities in health between the worse-off and the better-off [33].

## Ethics

This study uses secondary data collected from perpetual surveys. The datasets are anonymously coded with no individual identification identifiable by the user. The user has explicit authorization to use the datasets made available for analysis.

## Results

Table 1 exhibits that the study sample (the balanced panel) of this study was represented by a greater number of females. In addition, the proportion of the 31–60 age group was relatively higher and the proportion of the over 60 age group was substantially lower in the balanced panel. The distribution of respondents (study sample in the balanced panel) with self-perceived health status as good and not good was similar between study sample and RLMS-1994 datasets. The inflation adjusted adult equivalent household income (mean and median) was smaller for the respondents in the study sample and also the proportion of respondents with access to public service utilities was smaller in the study sample when compared with RLMS – 1994 datasets. However, the rural respondents and the respondents with having own tractor was higher in the study sample. Though the distribution of respondents by SES quintile (constructed) was almost similar between study sample and RLMS – 1994 datasets, the test of independence between the study sample and RLMS – 1994 datasets were significant for all the variables used in the study except for such test of respondents with having motor vehicle.

The descriptive statistics (Table 1) reflected a decline (from good to not good) in self-perceived health status by more than 20 % of the respondents over the 19 year period (1994 – 2013). This period registered a rise in diagnosed chronic disease by the respondents. During the same period affluence level (proxied by the ownership of the assets and savings by the household) and living condition (proxied by the access to the public service utilities) exhibited a positive movement amongst the respondents. The inflation adjusted adult equivalent household income (mean) increased by more than 85 % amongst the respondents from 1994 to 2013. The Gini index for the study sample changed from 0.272 in 1994 to 0.382 in 2013. But overall satisfaction in life increased by 2.3 times amongst the respondents of this study during the 19 years (1994 to 2013).

Table 2 presents the selection of study sample from RLMS – 1994 datasets. We found that study sample was a representation of more female respondents with relatively smaller household income and less house owners. There was no statistically significant difference in the self-perceived health status between the study sample and RLMS – 1994 datasets when other potential selection variables are included in the model. There was no gender – age interaction in the sample selection process.

**Table 2:** Logit model explaining sample [*N = 1496*] selection from RLMS datasets – 1994 [*N = 8893*]*

Variables	Coefficient
Self-perceived health (Comparison group: Very good)	
Good	0.47
Average	0.72
Bad	0.29
Very bad	0.28
Age	-0.01
Gender	0.78**
Age* Female (interaction term^a^)	0.00
Diagnosed chronic disease	-0.44*
Geography of residence (1 = urban)	-0.30*
Overall satisfaction with life (1 = satisfied)	-0.12
Adult equivalent household income	-0.07***
Ownership of assets	
Tractor (1 = yes)	0.27
Motor vehicle (1 = yes)	0.03
Country house (1 = yes)	-0.16
House (1 = yes)	-0.38**
Additional apartment (1 = yes)	-0.16
Living condition (access to public services utilities)	
Central heating	-0.24
Hot water supply	-0.16
Cooking gas	0.08
Central garbage disposal	0.26
Other indicators of economic position	
Having savings in the household	0.09
Grow crops (incl. Growing vegetables)	0.28
Constant	-2.32***
Pseudo R^2^	0.054

legend: *p < 05; **p < 0.01; ***p < 0.001

*The dependent variable is equal to 1 if respondents are included in the sample and 0, otherwise

^a^Interaction term captures gender effect on age

Table 3 indicated that the non-standardized perceived health gets worse over time, as one can expect. However, in our sample, the standardized perceived health improves over time. A predictable trend was observed – the non-standardized self-perceived health status declined while the standardized self-perceived health status improved over time. The test of significance (t-values) indicated that for the sample population, the standardized self-perceived health status from 2002 onwards and non-standardized self-perceived health status from 2006 onwards remained same as was in 2013.

**Table 3:** The mean of self-perceived health status (*N = 1496*)

Year	Self-perceived health status (mean)	Test of difference from 2013, t - test, ρ	Standardized self-perceived health status (mean)	Test of difference from 2013, t - test, ρ	Difference in mean (standardized - non-standardized) self-perceived health status
1994	2.88	0.000	3.06	0.000	0.18
1995	2.84	0.000	3.01	0.000	0.17
1996	2.84	0.000	2.99	0.005	0.15
1998	2.86	0.000	2.98	0.019	0.12
2000	2.90	0.000	2.97	0.033	0.07
2001	2.93	0.00	2.98	0.016	0.05
2002	2.92	0.01	2.96	0.136	0.03
2003	2.95	0.032	2.97	0.055	0.02
2004	2.93	0.006	2.94	0.589	0.00
2005	2.96	0.010	2.93	0.625	-0.02
2006	3.01	0.961	2.97	0.025	-0.04
2007	2.99	0.108	2.93	0.635	-0.06
2008	3.00	0.017	2.93	0.872	-0.07
2009	3.02	0.659	2.93	0.689	-0.09
2010	3.03	0.387	2.91	0.463	-0.12
2011	3.05	0.168	2.91	0.502	-0.14
2012	3.04	0.530	2.89	0.110	-0.16
2013	3.10	Comparison year	2.92	Comparison year	-0.17
Average	2.96		2.95		-0.01

CI was positive for non - standardized and standardized self-perceived health status for all the years (Table 4). The difference in absolute size between CI for non - standardized and that of standardized self-perceived health status was 0.149 in 1994 and 0.06 in 2013 i.e., a reduction of almost 60 % over the 19 year period. The annualized average of this difference was 0.052. A smaller standardized variant of CIs implied that some of the inequalities in self-perceived health status were unavoidable (attributable to the age structure of the sample). Though the CI for both the non - standardized and standardized self-perceived health status changed in every year, such change in absolute size for both the CIs did not follow any definite trend. A relatively smaller standard error for CIs with standardized self-perceived health status suggested that the serial correlation was corrected with the method of indirection standardization – standardization reduced the variations in self-perceived health status. The t-values indicated the existence of significant inequalities in self-perceived health status in almost all the years without any definite trend in changes of such inequalities. The weighted average of self-perceived health status [*I(v)*] was equal to CI when *v* was equal to 2*;* with increased *v* (increasing weight on the self-perceived health status of the worse-off) disachievement became significantly large enough for all the years. The highest disachievement was in year 2000.

**Table 4:** Levels of and inequalities* of self-perceived health status

	Self-pereceived health		Self-pereceived health - standardized				
					*v = 2*	*v = 3*	*v = 5*
Year	^a^ *C.I. [S.E.]*	^c^ *t-test(CI)*	^b^ *C.I. [S.E.]*	^c^ *t-test(CI)*	*I(v) [S.E.]*	*I(v) [S.E.]*	*I(v) [S.E.]*
2013	0.112 (0.034)	3.29	0.052 (0.019)	2.74	0.052 (0.019)	0.072 (0.028)	0.133 (0.041)
2012	0.153 (0.032)	4.78	0.043 (0.020)	2.15	0.043 (0.020)	0.068 (0.030)	0.149 (0.042)
2011	0.047 (0.031)	1.52	0.061 (0.019)	3.21	0.061 (0.019)	0.091 (0.030)	0.167 (0.041)
2010	0.071 (0.031)	2.29	0.067 (0.018)	3.72	0.067 (0.018)	0.107 (0.030)	0.198 (0.040)
2009	0.090 (0.032)	2.81	0.067 (0.017)	3.94	0.067 (0.017)	0.104 (0.026)	0.187 (0.038)
2008	0.136 (0.031)	4.39	0.072 (0.018)	4.00	0.072 (0.018)	0.118 (0.027)	0.221 (0.039)
2007	0.131 (0.030)	4.37	0.065 (0.017)	3.82	0.065 (0.017)	0.101 (0.026)	0.190 (0.038)
2006	0.144 (0.035)	4.11	0.059 (0.017)	3.47	0.059 (0.017)	0.104 (0.025)	0.212 (0.036)
2005	0.108 (0.031)	3.48	0.086 (0.018)	4.78	0.086 (0.018)	0.133 (0.027)	0.241 (0.038)
2004	0.125 (0.030)	4.17	0.053 (0.017)	3.12	0.053 (0.017)	0.08 (0.027)	0.157 (0.038)
2003	0.092 (0.029)	3.17	0.052 (0.017)	3.06	0.052 (0.017)	0.070 (0.027)	0.126 (0.039)
2002	0.065 (0.029)	2.24	0.067 (0.017)	3.94	0.067 (0.017)	0.106 (0.026)	0.199 (0.038)
2001	0.107 (0.028)	3.82	0.071 (0.018)	3.94	0.071 (0.018)	0.102 (0.027)	0.186 (0.038)
2000	0.153 (0.034)	4.50	0.101 (0.022)	4.59	0.101 (0.022)	0.151 (0.033)	0.262 (0.046)
1998	0.159 (0.032)	4.97	0.072 (0.021)	3.43	0.072 (0.021)	0.104 (0.033)	0.180 (0.049)
1996	0.135 (0.032)	4.22	0.068 (0.022)	3.09	0.068 (0.022)	0.083 (0.033)	0.133 (0.049)
1995	0.112 (0.033)	3.39	0.099 (0.020)	4.95	0.099 (0.020)	0.144 (0.032)	0.230 (0.046)
1994	0.201 (0.035)	5.74	0.052 (0.020)	2.60	0.052 (0.019)	0.072 (0.029)	0.130 (0.043)
Average	0.119 (0.032)		0.067 (0.019)		0.067 (0.019)	0.101 (0.029)	0.183 (0.041)

^a^for self-perceived health status (non-standardized)

^b^for self-perceived health status (standardized)

^c^calculated from the indices and standard error [47]

S.E. = standard error (with bootstrapped)

*All estimates are significant at *p* < 0.001

## Discussion

We followed 1,496 adults over a period of 19 years (1) to investigate the evolution of health differences among Russians in the context of neo-liberal restructuring with welfare-state retrenchment [34], and (2) to examine the distribution of evolved health differences across the socioeconomic strata of the population from 1994 to 2013. Our approach removed the effect of the changing composition of the sample and relied on the inter-individual differences to reduce the collinearity between current and lag variables to estimate unrestricted time-adjustment patterns [35]. Denisova [36] acknowledged potential importance of attrition bias in the study using RLMS datasets of 14 years (1994 – 2007) while concluding that the effect of such attrition bias did not have any significant health differences between those left in the sample and those remained in the sample datasets of RLMS. Further, applying inverse probability weighting (IPW) estimation method [37] on RLMS datasets (2001 – 2010), Gerry and Papadopoulos [38] have established that self-perceived health (SAH) related attrition in the longitudinal elements of RLMS surveys do not have the effect on the model results.

In spite of this fact, the individuals in the sample are getting older, and our results indicated a systematic trend of improvement, though not consistent, in the age-, gender-, and diagnosed chronic diseases-standardized mean of the self-perceived health status after controlling for the effect of region, settlement of residence (urban or rural), and SES for the study population. The standardized mean self-perceived health status registered an overall positive shift of 4.60 % from 1994 to 2013. During this period (1994–2013), the Gini index – a measure of distributional equality (0 = perfect equality; 1 = absolute inequality) of material affluence – became positive by 40.44 %. Our results also supported the evidence from Gerry and Papadopoulos [38] who had showed that there was a significant unobserved individual heterogeneity associated with age and with initial self-perceived health status in the RLMS datasets (2001 – 2010).

We could not find any obvious effect of the macroeconomic crisis (1998) on the self-perceived health status within our study cohort. Though inequality indices (the concentration index; a bivariate measure of inequality, measuring inequality in self-perceived health status related to the ranking of the individual within SES) in the distribution of good self-perceived health status (non-standardized) became smaller in 2013 compared to 1994 (a year that was economically weak for Russians) – suggesting a concentration of the good self-perceived health status (non-standardized) in the worse-off SES quintile in 2013 – no systematic trend emerges in the time path of concentration indices over almost two decades (1994–2013). The highest value of the disachievement index of year 2000 might be attributed to the macroeconomic effect of the 1998 economic crisis on standardized self-perceived health status of the study population.

This absence of consistent time trend is largely consistent with a similar phenomenon found in previous research [14] using Swiss survey datasets. A same absolute size of CI for standardized self-perceived health status in 1994, 2003, 2004 and 2013 indicated a uniform effect of age, gender and diagnosed chronic diseases on the self-perceived health status of the study population when effect of the confounding variables i.e., region, settlement of residence, and SES were controlled. Average effect of these standardising variables on the self-perceived health status of the study population was found to be more than 40 % but without any consistent trend on year-on-year. Perhaps a phenomenon like this can be explained by the pathways [10] from socioeconomic strata to health that have shaped individual responses to self-perceived health status. In addition, changes in concentration indices can be decoded as the expressions of macroeconomic factors on happiness (fluctuations in negative affect) when the economy is still dynamic, open and volatile [12]. Further, such a phenomenon also reflects life course variation in the SES gradient in health [39, 40].

Concerning the extent to which the inequality in good self-perceived health status varies with position of the individual in SES quintile, we found evidence that the variations of good self-perceived health status (both standardized and non-standardized) was not uniform within study population across. We found evidence that the better-off enjoys good self-perceived health status in all the years. Though the absolute size of CI for non - standardized self-perceived health status was reduced by 44.27 % from 1994 to 2013, there was not much change in the distribution of study population across SES quintiles in these two years but overall satisfaction with life for the study population became more than doubled from 1994 to 2013. Further, inequalities (difference between the CIs of non-standardized and standardized self-perceived health status) in self-perceived health status for the sample population was reduced by almost 60 % over the 19 year period. This reflection of positive shift of good health for the worse-off was consistent with plausible theoretical and empirical findings that the self-perceived health status of the individual is related to subjective rank perceptions of the individual with the SES [41–44], in other words, subjective socioeconomic status (SSS)^7^ rather than SES relates better to the self-perceived health status of the individual.

Marchand et al. [45] argues that any health gains among the better-off in the course of implementing efforts to improve the health of the worse-off is a positive externality because of the dilution in inequality reduction. Such argument translates to a policy that resulted in the same proportional improvement in everyone’s health would raise the value of the distributionally-sensitive measure of population health, while a policy that led to the same increase in the mean but a larger (smaller) proportional improvement in the health of the poor would produce a larger (smaller) increase in it. The mean self-perceived health status weighs everyone’s health equally, so, we estimated degree of aversion [33] to self-perceived health differences between the worse-off and the better-off within the study population. With the weights attached to the standardized self-perceived health status of the worse off (*v = 3 and v = 5),* we found disachievement (*I(v))* was becoming larger and larger, indicating that the values of the achievement index are sensitive to the weight set on the health of the worse-off population.

## Conclusion

An orientation towards inequity demands reduction of health status difference between worse-off (disadvantaged) and the better-off. The significance of measuring health inequality becomes a policy relevant tool if we can identify the aversion to inequality. The Gini coefficient, a general measure of the distribution of wealth, often does not correlate with the measure of health inequality (concentration index) in a transitional economy that lacks predictability. Furthermore, subjective socioeconomic status (SSS), proxied by overall satisfaction with life has emerged as an important determinant that correlates better with self-perceived health status. SSS, the rank-based facets of SES, shapes the revealed health status for the Russian Federation. We consider our results fairly robust, since we find the presence of diagnosed chronic disease risks in the respondents with bad and very bad self-perceived health.

This study contributes by exploring the evolution of differences in self-perceived health in the transition from a welfare regime to a market-driven open economy. We analysed the recent survey datasets to capture the changes and the distribution in self-perceived adult health status in different socioeconomic stages of life course. This panel study, which has followed the adult respondents for a long period (1994–2013), provides empirical evidence in subscribing to the fact that (1) the in-country average of self-perceived health status is not sufficient enough to guide policy action in reducing health inequalities, and (2) the socioeconomic strata-specific mean and the distribution of self-perceived health status within socioeconomic strata are important guides to improve the average health of the population.

Despite unfolding self-perceived health status gradient of selected individuals over 19 year period, the study sample selection and size of the study sample limits the generalisability of the study findings for the entire Russian population. RLMS is a nationally representative instrument with a multistage stratified sample, but the size of our cohort restricts the full representation of the entire country. The selection can have an impact on the association between neighbourhood factors and self-perceived health status. Though our measure of collective efficacy^8^ is purged of its association with individual-level social support and sociability, the datasets do not allow individual-level control to be derived directly from RLMS.

Our finding that differences in the distribution of self-perceived health cannot be explained by the socioeconomic strata position of the individual suggests that future research should take into account the context of stated self-perceived health status in the realm of subjective socioeconomic status (SSS), i.e., the individual’s subjective perceptions of their position relative to others in the socioeconomic hierarchy of the neighbourhood.

## Appendix - A

The concentration curve plots the cumulative proportion of self-perceived health (y) against the cumulative share of the population ranked by SES variables. The curve lies below the 45° line (diagonal) of equality if self-perceived health is concentrated among the better-off and above the 45° line (diagonal) of equality if self-perceived health is concentrated among the worse-off. The CI is defined as twice the area between the concentration curve and the diagonal (the line of equality).

$CI = \frac{2}{n\mu }{\displaystyle {\sum}_{i=1}^n{y}_i{R}_i-1}$, n is the sample size and R denotes the individual’s fractional rank (position of the individual) in the SES distribution. *μ*, the mean of the binary variable *y* (self-perceived health status) whose distribution across SES is the subject of interest. For, *μ* > 0 (if *y* = 0 for all *i*, CI is undefined) the minimum value of CI is equal to, $\mu -1+\left(\frac{1}{n}\right)$, and the maximum value is equal to $1-\mu +\left(\frac{1}{n}\right)$.

For a given, *μ* > 0, the maximum of the CI is when the poorest *j* individuals have a value of *y* equal to zero, and the richest *n* − *j* individuals have a value of *y* equal to one.

Therefore, $\mu =\frac{n-j}{n}$ and $\mathrm{C}\mathrm{I}=1-\mu +\frac{1}{n}$. For the large samples, the $\frac{1}{n}$ term vanishes, and the minimum and maximum tend to *μ* − 1 and 1 − *μ* respectively [46].

${R}_i={\displaystyle {\sum}_{j=1}^{i-1}{w}_j+\frac{1}{2}{w}_i}$, where *w*_0_ = 0. *R*_*i*_ denotes the weighted cumulative proportion of the population up to the midpoint of each individual weight and is bounded in the (0;1) interval. *R*_*i*_ represents the cumulative distribution function of SES and indicates the individual’s position within the SES distribution.

We estimated CI from regression of a transformation (correction of the standard error for across SES correlation owing to the rank nature of the regressor) of the self-perceived health status on the fractional rank in SES distribution [47]. Newey–West regression [48], which corrects for autocorrelation as well as heteroscedasticity,

## Appendix – B

Achievement index: $$ I(v)=\frac{1}{N}{\displaystyle {\sum}_{i=1}^N{\mathrm{h}}_iv{\left(1-{R}_i\right)}^{v-1}} $$

where,

- *h is a measure of standardized self-perceived health (the high value of I*(*v*) *is to be considered as good)*
- *v = inequality aversion parameter; if, v = 1, everyone’s health is weighted equally; as, v is raised above 1, the weight attached to the health of a very poor person rises*
- *R*_*i*_*= fractional rank of the individual; so, the weight attached to the i*^*th*^*person’s health share is, v*(1 − *R*_*i*_)^*v* − 1^*.*

Hence, the achievement index captures inequality in the distribution of health and is deduced to

$$ \begin{array}{l}I(v) = \mu \left(1-C(v)\right),\\ {}\left[C(v) = - \frac{v}{\mu }\ cov\left\langle {h}_i,\ {\left(1-{R}_i\right)}^{v-1}\right\rangle\ \right]\end{array} $$

If standardized self-perceived health declines monotonically with SES, the greater the degree of inequality aversion, the greater the wedge between the mean *μ* and the value of the index *I*(*v*).

## Footnotes

1 Because of the decline in response rate in big cities, the proportion of the big cities in the sample became less than needed and continued to decrease each round, so in Round 15 (2006; http://www.cpc.unc.edu/projects/rlms-hse/data) sample repair was done. New households were added to reconstruct the share of each region in the sample (to make it equal to that of 1994 sample).

2 OECD-modified scale. After having used the “old OECD scale” in the 1980s and the earlier 1990s, the Statistical Office of the European Union (EUROSTAT) adopted in the late 1990s the so-called “OECD-modified equivalence scale”. This scale, first proposed by Haagenars et al. (1994), assigns a value of 1 to the household head, of 0.5 to each additional adult member and of 0.3 to each child.

3 $G = \frac{2\  covar\left(y,\ {r}_y\right)}{N\overline{y}}$, where *covar*(*y*, *r*_*y*_) is the covariance between income (*y*) and ranks of all households according to the income (*r*_*y*_) ranging from the poorest household (rank = 1) to the richest (rank = N). N is the total number of households, and $\overline{y}$ is the mean of the adult equivalent household income (YitzhakI, 1994 and Lerman & Yitzhaki, 1984).

4 Indirectly standardised health is the difference between observed and expected health where expected health for an individual is the average health of individuals with the same levels of the standardising variables as the individual. With groups, expected health for an SES group is the weighted average of health levels conditional on the standardising variables, where the weights are the proportion of the SES group population in the sub groups defined by the standardising variables.

5 A regression method that corrects for heteroscedasticity and autocorrelation.

6 Defined as a weighted average of the self-perceived health status of the respondents where higher weights are attached to poorer people. It reflects the average level of self-perceived health status and the inequality in health (expressed as self-perceived health status) between the worse-off and the better-off.

7 Subjective socioeconomic status (SSS) is defined as a person’s subjective perceptions of their rank, relative to others, in the socioeconomic hierarchy (Kraus, Piff, & Keltner, 2011, 2009; Singh-Manoux, Adler, & Marmot, 2003 and Adler, Epel, Castellazo, & Ickovics, 2000).

8 Emphasizes mutual trust and solidarity (social cohesion), and shared expectations for pro-social action (informal social control) in theorizing the impact of neighborhood social organization on local residents’ well-being. Evidence suggests that collective efficacy is a generalizable resource capable of influencing a wide range of outcomes, including self-perceived (self-perceived) health status (Browning & Cagney, 2002).
