# Erratum to: Health inequality in the Russian federation: an examination of the changes in concentration and achievement indices from 1994 to 2013

**DOI:** 10.1186/s12939-016-0339-3

**Published:** 2016-03-18

**Authors:** Pavitra Paul, Hannu Valtonen

**Affiliations:** Department of Health and Social Management, Faculty of Social Sciences and Business Studies, University of Eastern Finland, Kuopio, 70211 Finland

Unfortunately, after publication of this article [1] it was noticed that there were two errors. The final line of the Background section should read, “Our data is unique in the sense that we follow the same individuals over an 18-year period.” The first line of the 4th paragraph of the Methods section should instead read, “Our dependent variable for the analysis is self-perceived (self-assessed) health (SAH).”
